# Supplementary material for: CRISPRs in the human genome are differentially expressed between malignant and normal adjacent to tumor tissue
Source: Commun Biol. 2022 Apr 8;5:338. doi: 10.1038/s42003-022-03249-4 (PMC8993844; doi:10.1038/s42003-022-03249-4)
Supplement: Supplementary file 2 — Supplementary Information [file 42003_2022_3249_MOESM2_ESM.pdf]

Supplementary Information for

CRISPRs in the human genome are differentially expressed between malignant and normal-adjacent to tumor tissue

Job van Riet [1,2,3¥], Chinmoy Saha [4¥], Nikolaos Strepis [4], Rutger W. W. Brouwer [5], Elena S. Martens-Uzunova [1], Wesley S. van de Geer [2,3], Sigrid M. A. Swagemakers [6], Andrew Stubbs [6], Yassir Halimi [4], Sanne Voogd [4], Arif Mohammad Tanmoy [4,7], Malgorzata A. Komor [8,9], Youri Hoogstrate [10], Bart Janssen [11], Remond J.A. Fijneman [8], Yashar S. Niknafs [12], Arul M. Chinnaiyan [12], Wilfred F.J. van IJcken [5], Peter J. van der Spek [6], Guido Jenster [1], Rogier Louwen [4*]

[1] Department of Urology, Erasmus MC Cancer Institute, University Medical Center Rotterdam, Rotterdam, Netherlands. [2] Cancer Computational Biology Center, Erasmus MC Cancer Institute, University Medical Center Rotterdam, Rotterdam, Netherlands. [3] Department of Medical Oncology, Erasmus MC Cancer Institute, University Medical Center Rotterdam, Rotterdam, Netherlands. [4] Department of Medical Microbiology and Infectious Diseases, Erasmus University Medical Center Rotterdam, Rotterdam, Netherlands. [5] Center for Biomics, Erasmus University Medical Center Rotterdam, Rotterdam, Netherlands. [6] Clinical Bioinformatics, Department of Pathology, Erasmus University Medical Center Rotterdam, Rotterdam, Netherlands. [7] Child Health Research Foundation, 23/2 SEL Huq Skypark, Block-B, Khilji Rd, Dhaka 1207, Bangladesh. [8] Translational Gastrointestinal Oncology, Department of Pathology, Netherlands Cancer Institute, Amsterdam, Netherlands. [9] Oncoproteomics Laboratory, Department of Medical Oncology, VU University Medical Center, Amsterdam, Netherlands. [10] Department of Neurology, Erasmus University Medical Center Rotterdam, Rotterdam, Netherlands. [11] GenomeScan, Leiden, Netherlands. [12] Michigan Center for Translational Pathology, University of Michigan, Ann Arbor, Michigan, USA

[¥] contributed equally to this work

Keywords: CRISPR-Cas, human, transcriptomics, small non-coding RNAs, repeats, disease, prostate cancer

* **Corresponding author** Email: r.louwen@erasmusmc.nl, Phone: 0031-(0) 6-50031638

This file includes:

Supplementary Notes
Supplementary Figures
Supplementary Tables
Supplementary Data
Supplementary References

Supplementary Note 1

In the early days CRISPR-Cas researchers were pioneering with computer-based methods to identify CRISPR arrays in the genomes of prokaryotes in a more standardized manner, which often included the Tandem Repeat Finder[1] and Locating Uniform poly-Nucleotide Areas software tools[2,3]. The first *in-silico* methods were relatively simple by using the sequence-similarity approach of BLASTN[4] to explore the presence of known repeat signatures in other organisms for which the genomes became available[5,6]. But, the inconsistency of CRISPR repeats between more distant bacterial species made it difficult to easily identify these signatures[6]. This led to the development and usage of a different software tool named PatScan[7], enabling the identification of sequence-specific CRISPR motifs in the EMBL/GenBank database, with a minor disadvantage that it could only identify CRISPR motifs with a minimum of four direct repeats in the genetic material deposited[6]. By using this software tool, CRISPR arrays were detected in approximately half of all analyzed bacterial genomes, but not in viral and eukaryotic genomes[8]. However, this software tool was still imperfect as it required the manual retrieval of the CRISPR arrays from the PatScan obtained sequences for further validation[8]. The identification of *cas* genes flanking the CRISPR arrays occurred via an independent alignment procedure using BLASTN and TBLASTN programs, revealing that the CRISPR-Cas system identification tools were still in its early days[8]. More elegant CRISPR software identification tools, such as, PYGRAM[9], REPfind[10], CRISPR recognition tool[11] and PILAR-CR[12] followed soon after, leading to a first visualization package for detected CRISPR arrays. Nevertheless, all these software tools still harbored their flaws leading to misidentifications of CRISPR arrays in the prokaryotic genomes[6]. In 2007, the CRISPRCasFinder tool was released with sophisticated identification and visualization software packages, such as the ability to detect short CRISPR-like structures (orphan CRISPR arrays) with or without flanking sequences containing the *cas* genes[6]. Spacer sequences could be extracted easily, enabling the ability to directly expose such sequences to BLAST searches, a real breakthrough in the CRISPR field at that time. Today, CRISPRCasFinder is often updated with new insights and features and is a well-accepted CRISPR identification tool[13]. Next to CRISPRCasFinder, two other identification tools have been published and released, named CRISPRCasTyper[14] and CRISPRDetect[15], which are relatively new and claim to address

the gaps still identified in CRISPRCasFinder. Indeed, it is argued that CRISPRDetect is more accurate and quantitatively superior in identifying CRISPR arrays when compared to CRISPRCasFinder[15], whereas CRISPRCasTyper can detect both the CRISPR arrays and their associated *cas* genes with increased accuracy[14]. Moreover, CRISPRCasTyper uses machine learning technologies to identify orphan and more distant arrays in metagenomic related datasets[14].

Supplementary Note 2

We compared the results of CRISPRDetect[15], CRISPRCasTyper[14] and the CRISPRCasFinder[13] software tools to each other by using the human reference genome (GRCh38). For CRISPRDetect this resulted in the identification of 3,653 hCRISPRs and 29 for CRISPRCasTyper (**Supplementary Data 2**). These analyses revealed that four hCRISPRs identified by the CRISPRCasTyper, were also identified by CRISPRDetect (*n* = 3) or CRISPRCasFinder (*n* = 1) (**Supplementary Data 2**). Of the hCRISPRs detected by CRISPRDetect (*n* = 3,653), 821 (23%) were also identified by CRISPRCasFinder (**Supplementary Data 2**). CRISPRMap was then used to identify specific characteristics of the CRISPR-Cas systems, including RNA structure motifs that fit known Cas protein cleavage sites in the CRISPR repeats, the presence of conserved repeat sequence families and superclasses[16]. Using CRISPRMap on the consensus repeat sequences retrieved from CRISPRCasTyper and CRISPRDetect resulted in the identification of specific RNA structure motifs that fit known Cas protein cleavage sites within the hCRISPR repeats (**Supplementary Data 2**). However, 13 consensus repeats detected by CRISPRDetect could also be linked to the repeat family six, 11 and 14 (**Supplementary Data 2**). On the other hand, analysis of the consensus repeats identified by CRISPRCasFinder, revealed a more complete landscape of repeats belonging to specific motifs, repeat family sequences and even superclasses (**Supplementary Data 2**). CRISPRMap is thus suited to identify unique and common features in the hCRISPR consensus repeat sequences, such as Cas endonucleases cleavage site motifs, conserved prokaryotic CRISPR repeat sequence families and repeat superclasses[16]**.** The overall outcome of the tool comparison is that CRISPRCasFinder identified by far the biggest number of hCRISPRs. Moreover, only the consensus repeats identified with CRISPRCasFinder could be linked to known repeat superclasses as present in CRISPRMap[16].

Then the CRISPRloci database[17] was used to identify whether known prokaryotic consensus repeats matched with the hCRISPR consensus repeats. The consensus repeats obtained from the hCRISPRs identified by CRISPRCasTyper did not result in any significant hits. However, the hCRISPR consensus repeats, which were identified with CRISPRDetect, revealed 21 CRISPR consensus repeats that harbored significant identity (p < 0.05) to the consensus CRISPR repeat as present in the CRISPRloci database. The significant hits included consensus CRISPR repeats identified in *Legionella*, *Geobacillus*, *Anoxybacillus*, *Campylobacter*, *Streptomyces*, *Staphylococcus* species, amongst others (**Supplementary Data 2**). Next, the CRISPRCasFinder detected consensus repeats revealed 59 hCRISPR consensus repeats that harbored significant identity (p < 0.05) to the consensus CRISPR repeat identified in *Enterococcus*, *Neisseria*, *Leptospira*, *Helicobacter*, *Anoxybacillus*, *Campylobacter*, *Streptomyces*, *Staphylococcus* species amongst others as deposited in the CRISPRloci database (**Supplementary Data 2**). Thus, some of the hCRISPRs harbor significant sequence similarity to CRISPRs as identified in other organisms, which we further investigated. We first assigned the hCRISPRs (n = 12,572) to specific phylum by sequence matching, which revealed that multiple organisms harbored a correlation in the six different kingdoms (**Supplementary Figure 3a**). Most of the assigned hCRISPRs were related to chordate animals, *Streptophyta* plants, *Artvervicota* viruses and proteobacteria. The proteobacterial overlap correlates well with the consensus repeat findings obtained with the previous CRISPRloci[17] results, which linked some of the consensus repeats to the proteobacteria *Neisseria*, *Campylobacter*, *Legionella*, *Helicobacter*, amongst others (**Supplementary Data 2**). Next, the hCRISPRs assigned to the chordate phylum were further evaluated, and interestingly, these resulted in the formation of multiple clusters with limited members (**Supplementary Figure 3b**). Only a few clusters were observed that contained ≥10 members of the hCRISPR (**Supplementary Figure 3b**). Overall, this low number of clusters indicates that a broad sequence diversity exists among the hCRISPRs. In addition to the hCRISPR identification, we also found during this analyses that nine of the hCRISPRs were accompanied with potential *cas* genes as revealed by CRISPRCasTyper[14], with one operon belonging to the Type I-C and another one belonging to the Type I-B CRISPR-Cas classification systems (**Supplementary Figure 3c**). The remaining seven candidate CRISPR-Cas operons were not classified (**Supplementary Figure 3c**). In line with the CRISPRCasTyper observation, CRISPRCasFinder[6] also revealed that some of the

hCRISPRs (*n* = 327) were accompanied with *cas* genes signatures. There against with the latest version of CRISPRCasFinder[13] we only identified two *cas* genes accompanying two hCRISPRs, named chr6_48 and chry_33 (**Supplementary Data 1**). The latter resides in gene *PCDH11Y* and is accompanied by *cas3*. Of note, *PCDH11Y* is a human gene involved in the development of nerve cells, is unique to males and likely jumped from chromosome X to Y about three million years ago[18,19]. The other *cas* gene identified is *cas2* and resides on an intergenic region in chromosome 6 and is accompanied by hCRISPR chr6_48. To exclude the possibility that the older version of the CRISPRCasFinder[6] misidentified domains in the coding genome that harbor high similarity to the domains present in the *cas* genes, we focused specifically on the non-coding genome. For 102 hCRISPRs in the non-coding genome this led to a more detailed analysis of their flanking regions, since CRISPRCasFinder[6] revealed potential *cas* gene signatures upstream or downstream of these hCRISPRs (**Supplementary Data 3**). By applying a 3D BLAST alignment, we identified that for 53 (52%) hCRISPRs the flanking regions harbored a LINE-1 retrotransposon element (**Supplementary Data 3**). From these 53 cases, 35 could also be linked by BLAST to an earlier reported reverse transcriptase *RVT_1* (PF00078) known of being fused to the *cas1* of *Marinomonas mediterranea*. The RVT_1-Cas1 of *Marinomonas mediterranea* enables the acquisition of spacer sequences from donor RNA[20] and thus is potentially capable of interfering with parasitic RNA sequences[20]. In our case, 23 of these 35 reverse transcriptase *RVT_1* (PF00078) signatures were also found to be accompanied with a *cas1* signature of *Marinomonas mediterranea* (**Supplementary Data 3**). Moreover, 15 of the 102 hCRISPRs within the non-coding regions of the human genome harbored multiple signatures of different *cas* genes in their flanking regions, including those of *cas2*, *cas9*, *cas12*, *cas13*, *cmr2* and *cmr3* (**Supplementary Data 3**). Also, remnants of toxin anti-toxin systems were identified by the presence of *vapB21* and *vapC* signatures (**Supplementary Data 3**). Solitary signatures of *cas* genes, such as a gene encoding for the PF09706 CRISPR protein family (Cas_CXXC_CXXC) and *csx3* were identified as well (**Supplementary Data 3**). Overall, 25 of the 35 *cas1* gene signatures identified by CRISPRCasFinder were confirmed by the 3D UniProt BLAST alignment (**Supplementary Data 3**). Interestingly, 34 out of the 35 CRISPRCasFinder identified *cas1* gene signatures could be linked to the presence of a LINE-1 retrotransposon element (**Supplementary Data 3)**. A more extensive analyses utilizing a *cas* gene repository published earlier[21,22] revealed that 4,893 out of 12,572 hCRISPRs

were accompanied with the reverse transcriptase family RVT_1 (PF00078) in their flanking regions (E-value of < 10^{-5}; **Supplementary Data 4**). Indeed, also in two of the nine CRISPR-Cas operons as identified with the CRISPRCasTyper software, the RVT_1 (PF00078) was identified at the boundary of an operon, of which one belonged to the Type 1-B CRISPR-Cas classification (**Supplementary Figure 3c**).

Supplementary Note 3

Comparing the different CRISPR-Cas identifying software tools revealed several important findings and concerns of our presented hCRISPRs. The number of hCRISPRs identified by CRISPRCasTyper was low, yet revealed a number of potential CRISPR-Cas operons in the human genome. The number of hCRISPRs identified with CRISPRDetect was increased compared with CRISPRCasTyper, but failed to identify complete CRISPR-Cas operons, whilst CRISPRCasFinder identified a multitude of hCRISPRs, of which some were accompanied with *cas* gene signatures. Only a few hCRISPRs were detected by all three of the CRISPR identification software tools, and only 23% were detected by CRISPRCasFinder and CRISPRDetect. This raises concerns about the robustness of the CRISPRCasFinder detected hCRISPRs, and works two ways. In other words, is CRISPRCasFinder under reporting or exaggerating the number of hCRISPRs? Subsequently, this uncertainty affects our analyses in this manuscript and will require further in-depth curation to deduce the true number of hCRISPRs. Off note, some of the hCRISPRs looked more like the repeat signatures as those described for the CRISPR repeats identified in the mitochondria of plants[23]. On the other hand, the CRISPRloci[17] and CRISPRMap[16] software tools and their databases provided more weight to our finding, in that sense that the consensus repeats identified with CRISPRCasFinder could be linked to known Cas endonucleases cleavage site motifs, conserved prokaryotic CRISPR repeat sequence families, and repeat superclasses as present in these databases. Further strengthening our discovery was the observation that the hCRISPRS could be assigned during our phylum analyses to the CRISPR arrays as present in the proteobacteria clade. Our CRISPRloci database analyses also came up with an identical result linking some of the hCRISPR consensus repeats to the proteobacteria *Neisseria*, *Campylobacter*, *Legionella*, *Helicobacter*, to name a few. We anticipate that our eukaryotic CRISPR results and the comparison of different CRISPR-Cas identification tools will help to further optimize the CRISPR-Cas identification software packages in their

ability to detect CRISPRs and potential *cas*-like genes beyond prokaryotes. Overall, a complex business in the CRISPR-Cas field, often leading to new surprises[24–29].

Next, CRISPR-Cas is an established adaptive defense system in bacteria and archaea, in which the *cas* genes are believed to be of significant importance in the adaptation and interference process[30,31]. However, our knowledge on these systems is rapidly expanding on roles other than defense[32], for example, endogenous gene regulation[33]. In the CRISPR-Cas field it is becoming increasingly accepted that orphan CRISPRs in bacteria and archaea exist[34–36], even ones with two repeats and one spacer[6,8,13,23,36,37], which can function independently of the *cas* genes in both defense and endogenous gene regulation[36,38–40]. Our finding that the hCRISPRs present themselves as orphan, in larger clusters or even as a complete CRISPR-Cas operon, feeds our suspicion that they might fulfill similar roles as seen in prokaryotes, which include functions such as endogenous gene regulation as reported in bacteria[41], chromosomal replication as reported in archaea[42], or even in adaptive immunity as reported for PIWI-interacting RNA-guided CRISPR-like immune responses in eukaryotes[43]. Whether the hCRISPRs are real CRISPRs, in that sense that they have evolved next to nuclease proteins to safeguard the human genome from foreign nucleic acids, as observed for CRISPR-Cas systems in prokaryotes[8] and PIWI-interacting RNAs in eukaryotes[43–46], requires further investigation. Excitingly, recent work by Zhang *et al*., suggests that such a functionality might exist in eukaryotes. They not only reported on the discovery that the transposons named IscB and TnpB might be ancestors of the CRISPR-Cas-related genes *cas9* and *cas12*, but also revealed that IscB and TnpB are accompanied with an active CRISPR array. Of importance, the Cas9 and Cas12 ancestor proteins were shown to harbor RNA-guided nuclease activity[27], suggestive that these eukaryotic CRISPR-Cas systems could play a role in defense as well. Our CRISPR findings were not limited to the human genome either, since we could extend our findings to other eukaryotes as based on our phylum analyses. More in line with this recent published work is our discovery that a substantial number of the hCRISPRs we identified in the non-coding genome were accompanied with LINE-1 transposons, transposase 22, transposon Tn916 and the reverse transcriptase RVT_1 (PF00078), all indicative for the presence of a mobile element, of which the latter has been shown to play roles in viral defense[47]. Indeed, *RVT_1* (PF00078) is known of being fused to *cas1* and their encoded proteins play roles in spacer acquisition, which is required for adaptive immunity features[48,49].

Next to that, the fusion of *RVT_1* and *cas1*, is also reported to be accompanied with *cas2*, *cas6* or *cas13*[48,49]. Our results indicated that *RVT_1* and *cas1* signatures in the non-coding regions of the human genome were also accompanied with *cas2* and *cas13* signatures. Other combinations were identified as well, with *RVT_1* being accompanied with *cas* gene signatures *cas5*, *cas8*, *cas9*, *cas12, cmr3*, among others, and toxin anti-toxin signatures such as *vapC* and *vapBC* that have been identified in CRISPR-Cas systems[50]. CRISPRCasTyper even revealed a complete CRISPR-Cas operon belonging to a CRISPR-Cas Type 1-B system being accompanied with RVT_1. Whether this is indicative that such systems have been obtained in the human genome by horizontal gene transfer, or that a *RVT_1-cas* module, by means of horizontal gene transfer[51], became combined with this Type 1-B system, remains to be elucidated.

Supplementary Figures

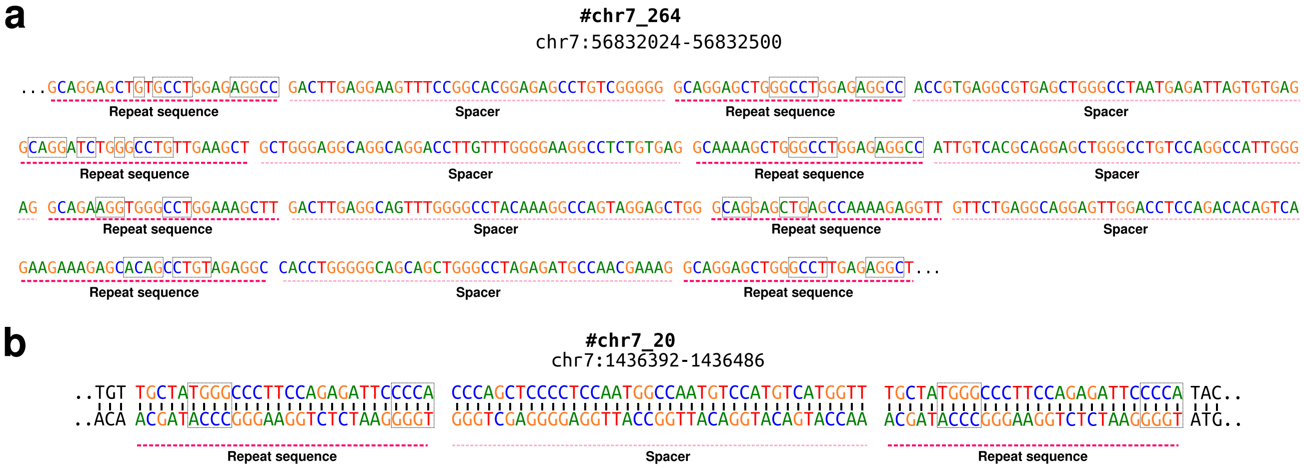

Supplementary Fig. 1, Overview of the sequence architecture of two typical hCRISPRs, highlighting the repeat, spacer and inner inverted repeat sequences.
Visualization of the hCRISPR sequence architecture for two different hCRISPRs as detected within the human reference genome (GRCh38). Repeat sequences are denoted by a thick dotted pink line whilst the subsequent spacer sequences are denoted by a narrow dotted pink line. The inverted repeat sequences, within the repeats, which enable the palindrome are highlighted with dotted black/grey boxes. Flanking up-/downstream sequences are shown as black nucleotide sequences bordering the hCRISPR. **a)** hCRISPR architecture for #chr7_264 and **b)** #chr7_20.

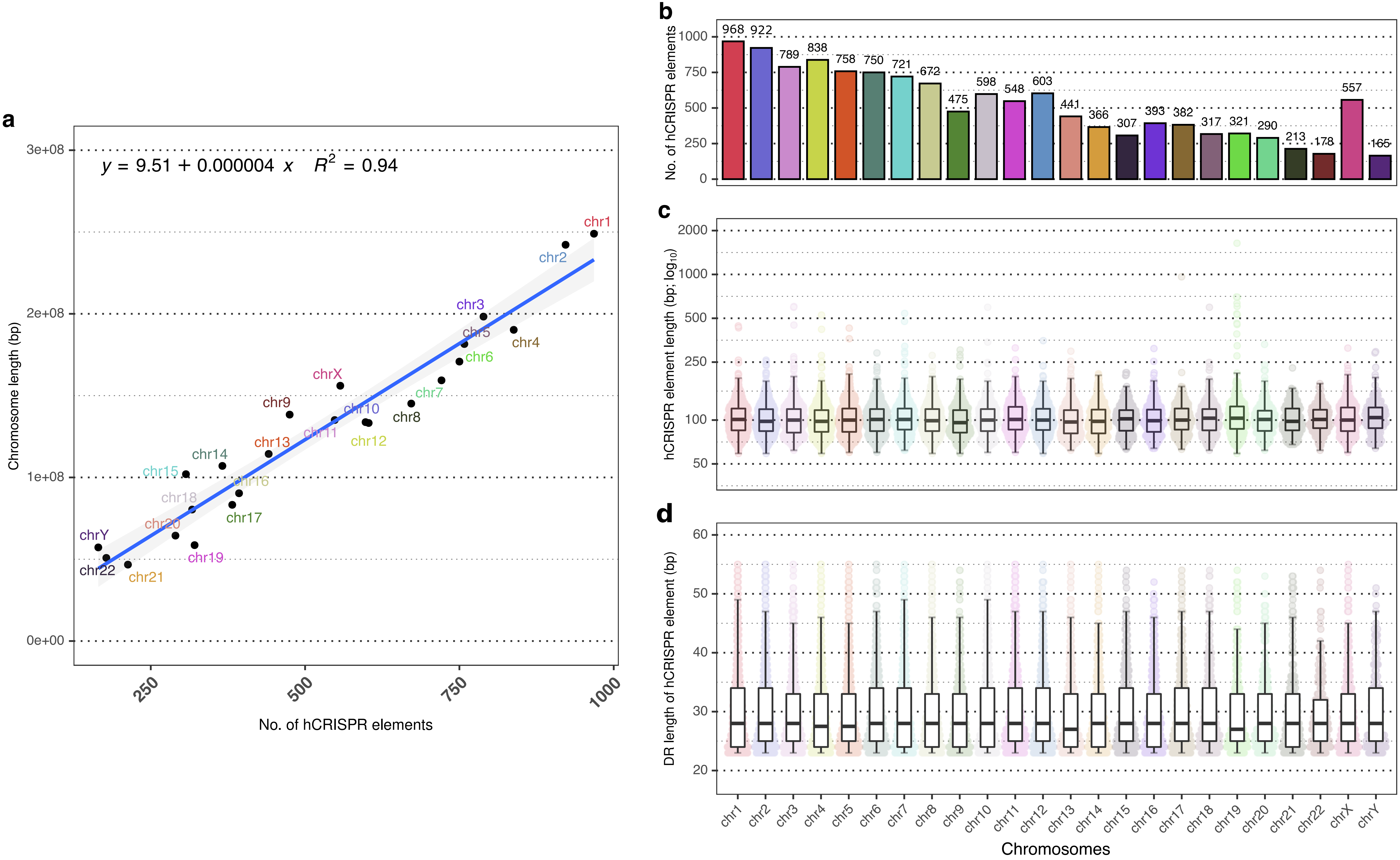

Supplementary Fig. 2, Characteristics of the hCRISPR nucleotide sequences.

a) Scatterplot with a linear model (x~y), with standard errors shown as transparent background, comparing chromosome lengths against the number of hCRISPRs present in that chromosome. The y-axis shows the chromosome length in base pairs (bp) and the x-axis reveals the number of detected hCRISPRs. The regression analysis (R^2 = 0.94) reveals that there is a correlation between the number of detected hCRISPRs and the length of the human chromosomes.

b) Total number of detected hCRISPRs per human autosomal and sex chromosome.

c) Boxplots representing the hCRISPR length (bp; $\log^{10}$) per human autosomal and sex chromosome; median, Q1 and Q3 are highlighted with a bold black line and error bars, respectively.

d) Boxplots representing the size of the direct repeat sequences in base pairs (bp) of the hCRISPRs per human autosomal and sex chromosome; median, Q1 and Q3 are highlighted with a bold black line and error bars, respectively.

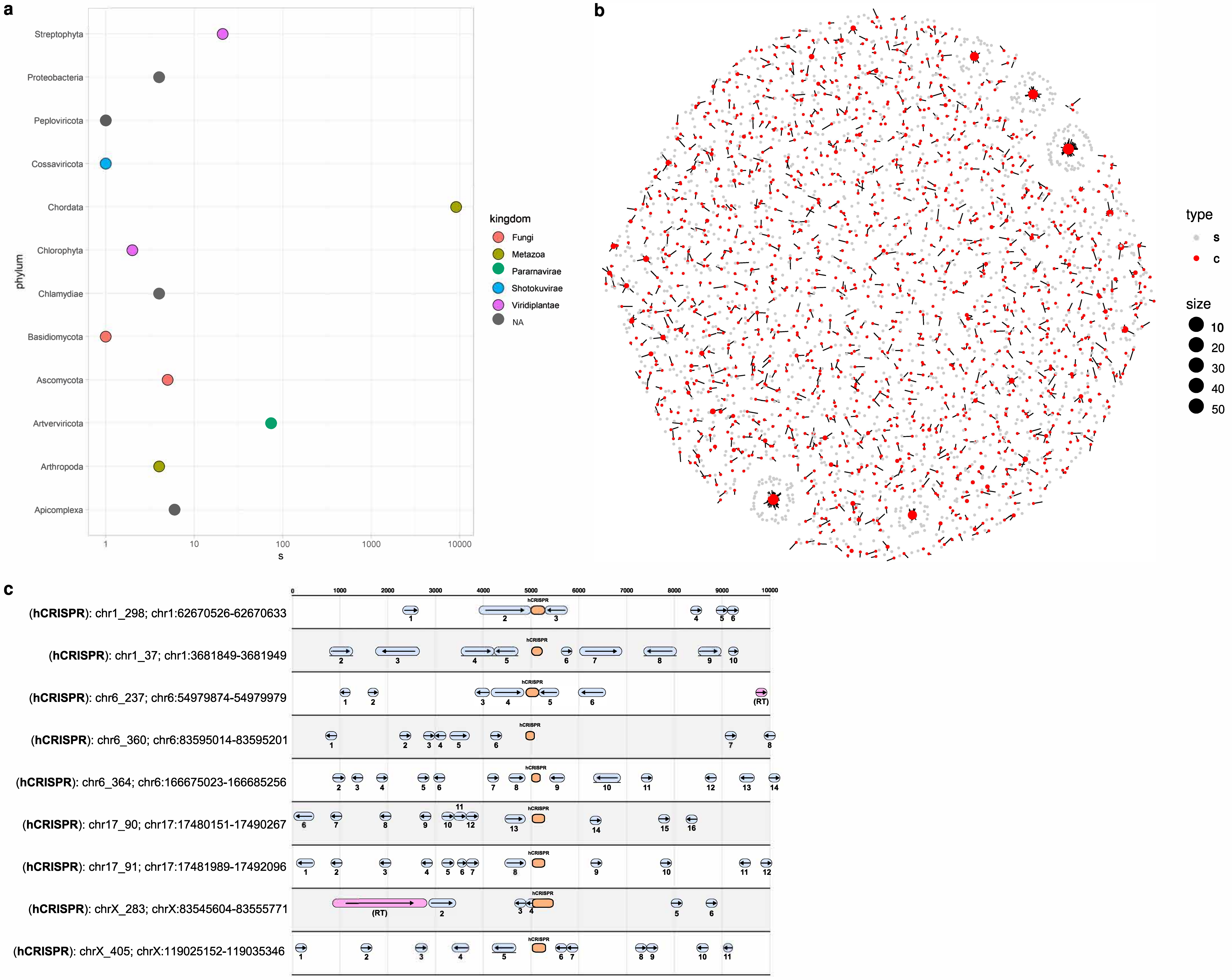

Supplementary Fig. 3, The hCRISPRs and their association with other organisms.

a) Assignment of the hCRISPRs (*n* = 12,572) to different phyla and kingdoms with S showing the number of hCRISPRs on the x-axis and the phylum on the y-axis.

b) Clustering (designated by type c) of the hCRISPRs as present in the Chordata phylum based on sequence similarities and the size of the clusters (black filled circles).

c) Schematic overview of the genomic distance for CRISPRCasTyper-detected CRISPR-Cas operons (numbered and orientation depicted by arrow) and reverse transcriptase (RT) genes (pink) surrounding two of the nine hCRISPRs (shown in orange).

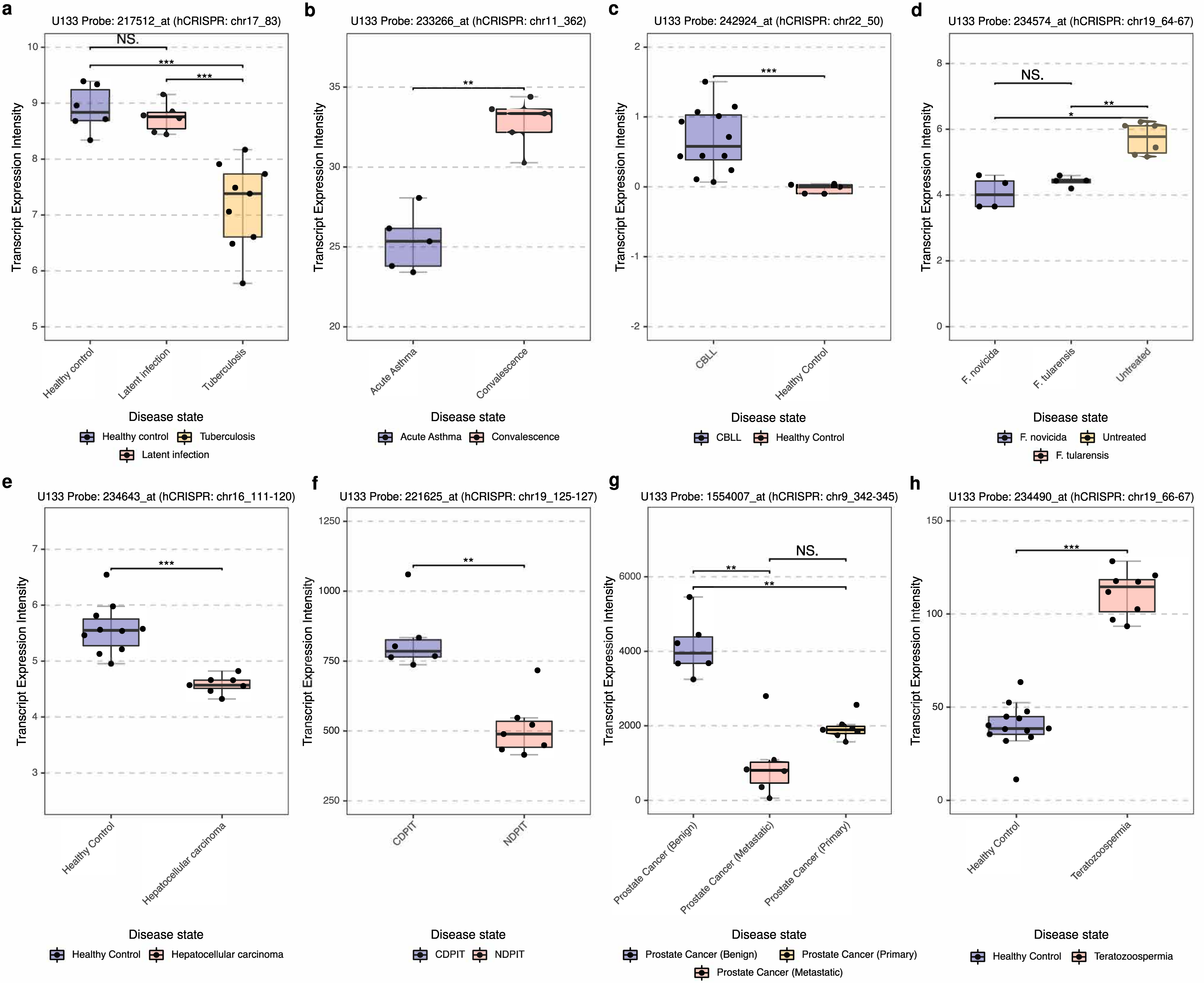

Supplementary Fig. 4, Disease-specific transcription of U133 probes overlapping hCRISPRs.
For each pairwise comparison, a two-sided Wilcoxon Signed Rank test was used to test statistical significance; $p > 0.05$ (NS.), $p \leq 0.05$ (*), $p \leq 0.01$ (**), $p \leq 0.001$ (***). Data is shown as boxplots with the median, Q1 and Q3 being highlighted with a bold black line and error bars, respectively.

a) U133 probe (217152_at) overlapping with hCRISPR (#chr17_83) shows potential in differentiating healthy controls ($n = 6$), latent Tuberculosis infected patients ($n = 6$) and patients displaying Tuberculosis ($n = 9$).

b) U133 probe (233266_at) overlapping with hCRISPR (#chr11_362) shows potential in differentiating convalescence ($n = 5$) from acute asthma patients ($n = 5$).

c) U133 probe (242924_at) overlapping with hCRISPR (#chr22_50) shows potential in differentiating healthy ($n = 5$) from chronic B-lymphocytic leukemia patients ($n = 12$).

d) U133 probe (234574_at) overlapping with hCRISPR (#chr19_64 to #chr19_67) shows potential in differentiating untreated blood monocytes ($n = 6$) compared to the *Francisella* species infected blood monocytes ($n = 8$).

e) U133 probe (234643_x_at) overlapping with hCRISPR (#chr16_111 to #chr16_120) shows potential in differentiating blood monocytes obtained from healthy controls ($n = 10$) compared to blood monocytes obtained from hepatocellular carcinoma (HCC) patients (n = 7).

f) U133 probe (221625_at) overlapping with hCRISPR (#chr19_125 to #chr19_127) shows potential in differentiating peripheral blood T-cells obtained from children with chronic ITP (CDPIT) ($n = 6$) compared to peripheral blood T-cells obtained from children with newly diagnosed ITP (NDPIT) ($n = 7$).

g)U133 probe (1554007_at) overlapping with hCRISPRs (#chr9_342 to #chr9_345) shows potential in differentiating prostate tumor tissue that was metastatic ($n = 6$), primary ($n = 7$) or benign ($n = 6$).

h) U133 probe (234490_at) overlapping with hCRISPR (#chr19_66 and #chr19_67) shows potential in differentiating teratozoospermia sperm cells ($n = 8$) compared to normal healthy sperm cells ($n = 13$).

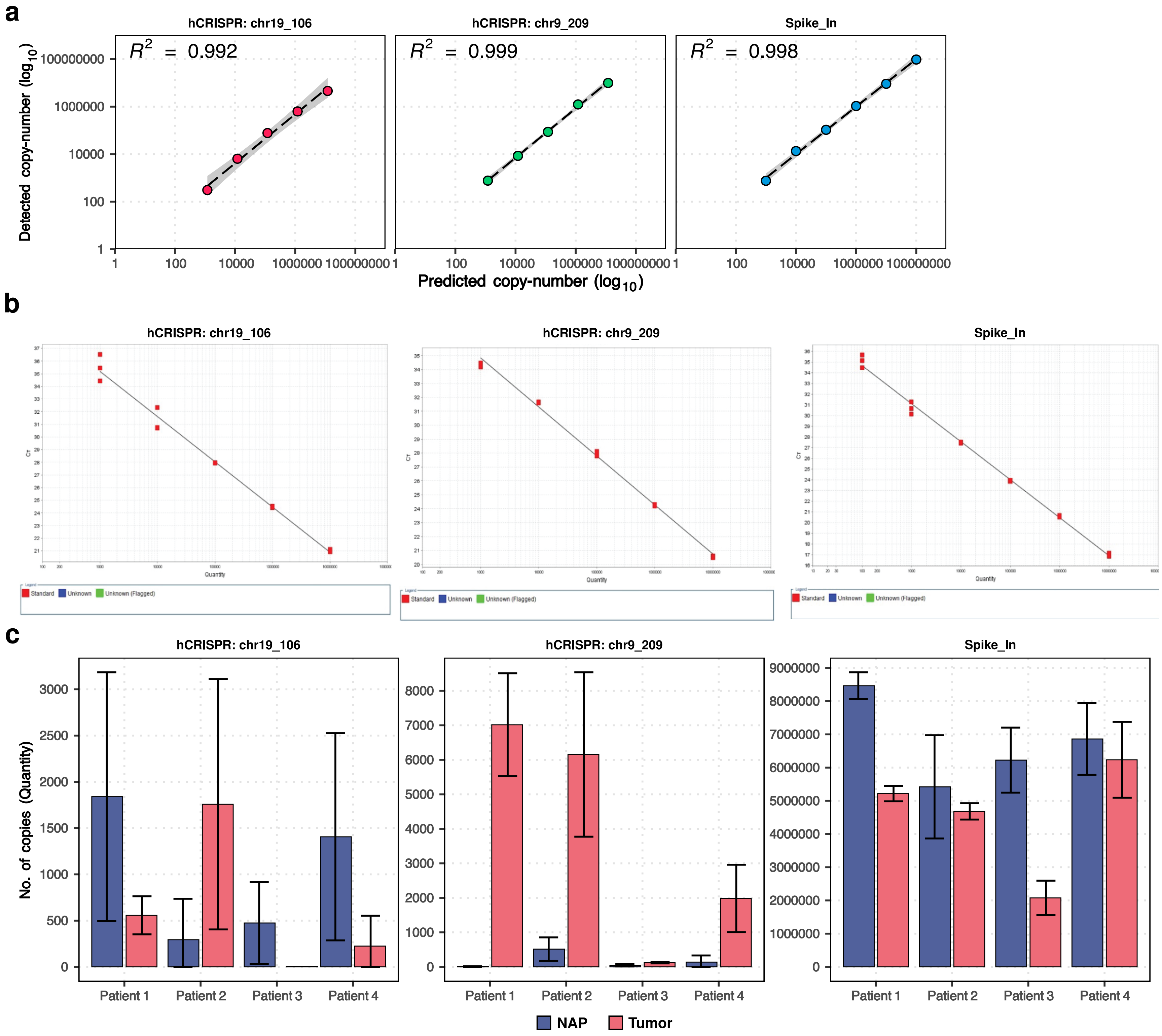

Supplementary Fig. 5, RT-qPCR assay copy numbers and standard curves.

a) A linear regression model of the correlation between the predicted transcript copy number (x-axis) and the detected copy number (y-axis) is plotted as a dotted line with the confidence interval shown as grey background. The R^2 of the linear regression is shown within the respective figures. Both axes are in $\log_{10}$ space.

b) Standard curves of chr19_106, chr9_209, and spike_In templates were prepared by 10-fold serial dilutions of an equimolar mix of chr19_106 and chr9_209 synthetic RNA templates and a spike_In control RNA.

c) Absolute copy numbers of chr19-106 and chr9-209 in patient samples measured by quantitative real-time PCR and determined by the standard curves in (**b**). The mean copy number values calculated from three technical replicates are depicted whilst error bars show the standard deviation. Normal adjacent-to -tumor tissue (NAP) and malignant tissue are colored in blue and pink, respectively.

Supplementary Tables

Supplementary Table 1 Standard curve parameters for each hCRISPR target detected in the RT-qPCR assay.

	Slope	Y-Inter	R2	Eff%
**Chr19_106**	-3.568	45.881	0.988	90.65
**Chr9_209**	-3.524	45.405	0.996	92.192
**Spike-In**	-3.558	41.79	0.996	91.024

Supplementary Data

Supplementary Data 1 hCRISPRs as detected by the CRISPRCasFinder software with genome location (hg38/GRCh38 lift over to hg19/GRCh37) combined with tissue, cell line and (pan-)cancer expression data and overlap with ENCODE tracks, genes, known repeats, transcription factor binding sites, DNase I Hypersensitivity Clusters, CpG methylation islands, piRBase, POLYAR, DASHR 2.0, RNAcentral, Erasmus MC PCa-associated transcripts better known as EPCATS, Bodymap expression and U133 plus 2.0 probes.

Supplementary Data 2 CRISPRMap and CRISPRloci findings after using the consensus repeats identified with the CRISPRCasFinder, CRISPRDetect and CRISPRCasTyper tools.

Supplementary Data 3 BLAST analyses in Expasy UniProt with a 3D structure feature of the flanking regions of the hCRISPRs.

Supplementary Data 4 BLAST analyses using the *cas*-gene repository aligned against the flanking regions of the hCRISPRs.

Supplementary Data 5 Overlap of the hCRISPRs with ENCODE transcription factor binding sites, DNase I Hypersensitivity Clusters and CpG methylation islands.

Supplementary Data 6 Detection of DASHR 2.0 sncRNAs originating from the 12,572 hCRISPRs by using the DASHR 2.0 UCSC Genome browser hub.

Supplementary Data 7 Annotated and un-annotated DASHR 2.0 sncRNA obtained from the four databases DASHR1, DASHR2, ENCODE GEO and ENCODE data portal that originated from the hCRISPRs.

Supplementary Data 8 Unannotated DASHR 2.0 sncRNAs that originated from the hCRISPRs which are SPAR confirmed.

Supplementary Data 9 hCRISPRs overlapping Mitranscriptome transcripts and showing their diagnostic potential.

Supplementary Data 10 U133 plus 2 hCRISPR related probes and disease detection that are visualized in **Supplementary Figure 4**.

Supplementary Data 11 hCRISPRs overlapping or exactly matching the U133 plus 2 probe regions or the used probes themselves and related additional diseases that showed diagnostic potential for a wide variety of diseases.

Supplementary Data 12 Data on the quantity of chr9_209, chr19_106 from the whole-transcriptome dataset (NGS-ProToCoL) and from the qPCR RNA-templates and assay linearity, including the synthetic spike_in.

Supplementary References

1. Benson, G. Tandem repeats finder: a program to analyze DNA sequences. *Nucleic Acids Res.* **27**, 573–580 (1999).

2. Peng, X. *et al*. Genus-specific protein binding to the large clusters of DNA repeats (short regularly spaced repeats) present in *Sulfolobus* genomes. *J. Bacteriol.* (2003) doi:10.1128/JB.185.8.2410-2417.2003.

3. Lillestøl, R. K., Redder, P., Garrett, R. A. & Brügger, K. A putative viral defence mechanism in archaeal cells. *Archaea* **2**, 59–72 (2006).

4. Altschul, S. F., Gish, W., Miller, W., Myers, E. W. & Lipman, D. J. Basic local alignment search tool. *J. Mol. Biol.* **215**, 403–410 (1990).

5. Haft, D. H., Selengut, J., Mongodin, E. F. & Nelson, K. E. A guild of 45 CRISPR-associated (Cas) protein families and multiple CRISPR-Cas subtypes exist in prokaryotic genomes. *PLoS Comput. Biol.* (2005) doi:10.1371 /journal.pcbi.0010060.

6. Grissa, I., Vergnaud, G. & Pourcel, C. CRISPRFinder: a web tool to identify clustered regularly interspace short palindromic repeats. *Nucleic Acids Res.* **35**, 52–57 (2007).

7. Dsouza, M., Larsen, N. & Overbeek, R. Searching for patterns in genomic data. *Trends Genet.* **13**, 497–498 (1997).

8. Jansen, R., Embden, J. D., Gaastra, W. & Schouls, L. M. Identification of genes that are associated with DNA repeats in prokaryotes. *Mol Microbiol* **43**, 1565–1575 (2002).

9. Durand, P., Mahé, F., Valin, A.-S. & Nicolas, J. Browsing repeats in genomes: Pygram and an application to non-coding region analysis. *BMC Bioinformatics* **7**, 477 (2006).

10. Betley, J. N., Frith, M. C., Graber, J. H., Choo, S. & Deshler, J. O. A ubiquitous and conserved signal for RNA localization in chordates. *Curr. Biol.* **12**, 1756–1761 (2002).

11. Bland, C. *et al.* CRISPR Recognition Tool (CRT): a tool for automatic detection of clustered regularly interspaced palindromic repeats. *BMC Bioinformatics* **8**, 209 (2007).

12. Edgar, R. C. PILER-CR: fast and accurate identification of CRISPR repeats. *BMC Bioinformatics* **8**, 18 (2007).

13. Couvin, D. *et al.* CRISPRCasFinder, an update of CRISRFinder, includes a portable version, enhanced performance and integrates search for Cas proteins. *Nucleic Acids Res.* (2018) doi:10.1093/nar/gky425.

14. Russel, J., Pinilla-Redondo, R., Mayo-Muñoz, D., Shah, S. A. & Sørensen, S. J. CRISPRCasTyper: Automated identification, annotation, and classification of CRISPR-Cas loci. *Cris. J.* **3**, 462–469 (2020).

15. Biswas, A., Staals, R. H. J., Morales, S. E., Fineran, P. C. & Brown, C. M. CRISPRDetect: A flexible algorithm to define CRISPR arrays. *BMC Genomics* **17**, 356 (2016).

16. Lange, S. J., Alkhnbashi, O. S., Rose, D., Will, S. & Backofen, R. CRISPRmap: an automated classification of repeat conservation in prokaryotic adaptive immune systems. *Nucleic Acids Res.* **41**, 8034–8044 (2013).

17. Alkhnbashi, O. S. *et al.* CRISPRloci: comprehensive and accurate annotation of CRISPR-Cas systems. *Nucleic Acids Res.* **49**, W125–W130 (2021).

18. Bachtrog, D. Y-chromosome evolution: emerging insights into processes of Y-chromosome degeneration. *Nat. Rev. Genet.* **14**, 113–124 (2013).

19. Mendez, F. L., Poznik, G. D., Castellano, S. & Bustamante, C. D. The divergence of neandertal and modern human Y chromosomes. *Am. J. Hum. Genet.* **98**, 728–734 (2016).

20. Silas, S. *et al.* Direct CRISPR spacer acquisition from RNA by a natural reverse transcriptase-Cas1 fusion protein. *Science* **351**, aad4234 (2016).

21. Makarova, K. S., Wolf, Y. I. & Koonin, E. V. Classification and Nomenclature of CRISPR-Cas Systems: Where from Here? *Cris. J.* **1**, 325–336 (2018).

22. Makarova, K. S., Anantharaman, V., Grishin, N. V, Koonin, E. V & Aravind, L. CARF and WYL domains: ligand-binding regulators of prokaryotic defense systems. *Front. Genet.* **5**, 102 (2014).

23. Mojica, F. J. M., Díez-Villaseñor, C., Soria, E. & Juez, G. Biological significance of a family of regularly spaced repeats in the genomes of Archaea, Bacteria and mitochondria. *Molecular Microbiology* vol. 36 244–246 (2000).

24. Burstein, D. *et al.* New CRISPR-Cas systems from uncultivated microbes. *Nature* **542**, 237–241 (2017).

25. van Beljouw, S. P. B. *et al.* The gRAMP CRISPR-Cas effector is an RNA endonuclease complexed with a caspase-like peptidase. *Science* **373**, 1349–1353 (2021).

26. Levasseur, A. *et al.* MIMIVIRE is a defence system in mimivirus that confers resistance to virophage. *Nature* **531**, 249–252 (2016).

27. Altae-Tran, H. *et al.* The widespread IS200/IS605 transposon family encodes diverse programmable RNA-guided endonucleases. *Science* **374**, 57–65 (2021).

28. Al-Shayeb, B. *et al.* Clades of huge phages from across Earth's ecosystems. *Nature* **578**, 425–431 (2020).

29. Doron, S. *et al.* Systematic discovery of antiphage defense systems in the microbial pangenome. *Science* **359**, (2018).

30. Westra, E. R., Buckling, A. & Fineran, P. C. CRISPR-Cas systems: beyond adaptive immunity. *Nat Rev Microbiol* **12**, 317–326 (2014).

31. Barrangou, R. *et al.* CRISPR provides acquired resistance against viruses in prokaryotes. *Science* **315**, 1709–1712 (2007).

32. Louwen, R., Staals, R. H. J., Endtz, H. P., van Baarlen, P. & van der Oost, J. The role of CRISPR-Cas systems in virulence of pathogenic bacteria. *Microbiol. Mol. Biol. Rev.* **78**, 74–88 (2014).

33. Sampson, T. R. & Weiss, D. S. Alternative roles for CRISPR-Cas Systems in bacterial pathogenesis. *PLoS Pathog.* (2013) doi:10.1371/journal.ppat.1003621.

34. Koonin, E. V. & Makarova, K. S. Origins and evolution of CRISPR-Cas systems. *Philosophical Transactions of the Royal Society B: Biological Sciences* (2019) doi:10.1098/rstb.2018.0087.

35. Makarova, K. S. *et al.* An updated evolutionary classification of CRISPR-Cas systems. *Nat. Rev. Microbiol.* (2015) doi:10.1038/nrmicro3569.

36. Almendros, C., Guzmán, N. M., García-Martínez, J. & Mojica, F. J. M. Anti-cas spacers in orphan CRISPR4 arrays prevent uptake of active CRISPR-Cas I-F systems. *Nat. Microbiol.* (2016) doi:10.1038/nmicrobiol.2016.81.

37. Jansen, R., van Embden, J. D. A., Gaastra, W. & Schouls, L. M. Identification of a novel family of sequence repeats among prokaryotes. *Omi. A J. Integr. Biol.* (2002) doi:10.1089/15362310252780816.

38. Mandin, P., Geissmann, T., Cossart, P., Repoila, F. & Vergassola, M. Identification of new non-coding RNAs in *Listeria monocytogenes* and prediction of mRNA targets. *Nucleic Acids Res.* (2007) doi:10.1093/nar/gkl1096.

39. Stern, A., Keren, L., Wurtzel, O., Amitai, G. & Sorek, R. Self-targeting by CRISPR: Gene regulation or autoimmunity? *Trends in Genetics* (2010) doi:10.1016/j.tig.2010.05.008.

40. Al-Shayeb, B. *et al.* Clades of huge phage from across Earth's ecosystems. *bioRxiv* 572362 (2019) doi:10.1101/572362.

41. Hille, F. & Charpentier, E. CRISPR-Cas: biology, mechanisms and relevance. *Philos. Trans. R. Soc. B Biol. Sci.* **371**, 20150496 (2016).

42. Mojica, F. J. M. M., Ferrer, C., Juez, G. & Rodríguez-Valera, F. Long stretches of short tandem repeats are present in the largest replicons of the Archaea *Haloferax mediterranei* and *Haloferax volcanii* and could be involved in replicon partitioning. *Mol. Microbiol.* **17**, 85–93 (1995).

43. Ophinni, Y., Palatini, U., Hayashi, Y. & Parrish, N. F. piRNA-Guided CRISPR-like Immunity in Eukaryotes. *Trends in Immunology* (2019) doi:10.1016/j.it.2019.09.003.

44. Grimson, A. *et al.* Early origins and evolution of microRNAs and PIWI-interacting RNAs in animals. *Nature* (2008) doi:10.1038/nature07415.

45. Shah, S. A. & Garrett, R. A. CRISPR-Cas and Cmr modules, mobility and evolution of adaptive immune systems. *Res. Microbiol.* (2011) doi:10.1016/j.resmic.2010.09.001.

46. Swarts, D. C. *et al*. The evolutionary journey of Argonaute proteins. *Nature Structural and Molecular Biology* (2014) doi:10.1038/nsmb.2879.

47. Steczkiewicz, K., Prestel, E., Bidnenko, E. & Szczepankowska, A. K. Expanding diversity of firmicutes single-strand annealing proteins: A putative role of bacteriophage-host arms race. *Front. Microbiol.* **12**, 644622 (2021).

48. Toro, N., Mestre, M. R., Martínez-Abarca, F. & González-Delgado, A. Recruitment of reverse transcriptase-Cas1 fusion proteins by Type VI-A CRISPR-Cas systems. *Front. Microbiol.* **10**, 2160 (2019).

49. Mohr, G. *et al*. A Reverse Transcriptase-Cas1 fusion protein contains a Cas6 domain required for both CRISPR RNA biogenesis and RNA spacer acquisition. *Mol. Cell* **72**, 700-714.e8 (2018).

50. Garrett, R. A., Vestergaard, G. & Shah, S. A. Archaeal CRISPR-based immune systems: exchangeable functional modules. *Trends Microbiol.* **19**, 549–556 (2011).

51. Silas, S. *et al*. On the origin of reverse transcriptase-using CRISPR-Cas systems and their hyperdiverse, enigmatic spacer repertoires. *MBio* **8**, (2017).
