# Supplementary material for: CRISPRs in the human genome are differentially expressed between malignant and normal adjacent to tumor tissue
Source: Commun Biol. 2022 Apr 8;5:338. doi: 10.1038/s42003-022-03249-4 (PMC8993844; doi:10.1038/s42003-022-03249-4)
Supplement: Supplementary file 3 — Description of Additional Supplementary Files [file 42003_2022_3249_MOESM3_ESM.pdf]

# Description of Additional Supplementary Files

**File name:** Supplementary Data 1
**Description:** hCRISPRs as detected by the CRISPRCasFinder software with genome location (hg38/GRCh38 lift over to hg19/GRCh37) combined with tissue, cell line and (pan-)cancer expression data and overlap with ENCODE tracks, genes, known repeats, transcription factor binding sites, DNase I Hypersensitivity Clusters, CpG methylation islands, piRBase, POLYAR, DASHR 2.0, RNAcentral, Erasmus MC PCa-associated transcripts better known as EPCATS, Bodymap expression and U133 plus 2.0 probes.

**File name:** Supplementary Data 2
**Description:** CRISPRMap and CRISPRloci findings after using the consensus repeats identified with the CRISPRCasFinder, CRISPRDetect and CRISPRCasTyper tools.

**File name:** Supplementary Data 3
**Description:** BLAST analyses in Expasy UniProt with a 3D structure feature of the flanking regions of the hCRISPRs.

**File name:** Supplementary Data 4
**Description:** BLAST analyses using the cas-gene repository aligned against the flanking regions of the hCRISPRs.

**File name:** Supplementary Data 5
**Description:** Overlap of the hCRISPRs with ENCODE transcription factor binding sites, DNase I Hypersensitivity Clusters and CpG methylation islands.

**File name:** Supplementary Data 6
**Description:** Detection of DASHR 2.0 sncRNAs originating from the 12,572 hCRISPRs by using the DASHR 2.0 UCSC Genome browser hub.

**File name:** Supplementary Data 7
**Description:** Annotated and un-annotated DASHR 2.0 sncRNA obtained from the four databases DASHR1, DASHR2, ENCODE GEO and ENCODE data portal that originated from the hCRISPRs.

**File name:** Supplementary Data 8
**Description:** Unannotated DASHR 2.0 sncRNAs that originated from the hCRISPRs which are SPAR confirmed.

**File name:** Supplementary Data 9
**Description:** hCRISPRs overlapping Mitranscriptome transcripts and showing their diagnostic potential.

**File name:** Supplementary Data 10
**Description:** U133 plus 2 hCRISPR related probes and disease detection that are visualized in Supplementary Figure 4.

**File name:** Supplementary Data 11
**Description:** hCRISPRs overlapping or exactly matching the U133 plus 2 probe regions or the used probes themselves and related additional diseases that showed diagnostic potential for a wide variety of diseases.

**File name:** Supplementary Data 12

**Description:** Data on the quantity of chr9_209, chr19_106 from the whole-transcriptome dataset (NGS-ProToCoL) and from the qPCR RNA-templates and assay linearity, including the synthetic spike_in.
