# Supplementary material for: CRISPRs in the human genome are differentially expressed between malignant and normal adjacent to tumor tissue
Source: Commun Biol. 2022 Apr 8;5:338. doi: 10.1038/s42003-022-03249-4 (PMC8993844; doi:10.1038/s42003-022-03249-4)
Supplement: Supplementary file 16 — Reporting Summary [file 42003_2022_3249_MOESM16_ESM.pdf]

nature portfolio

Corresponding author(s): Dr Rogier Louwen

Last updated by author(s): Feb 22, 2022

Reporting Summary

Nature Portfolio wishes to improve the reproducibility of the work that we publish. This form provides structure for consistency and transparency in reporting. For further information on Nature Portfolio policies, see our Editorial Policies and the Editorial Policy Checklist.

Statistics

For all statistical analyses, confirm that the following items are present in the figure legend, table legend, main text, or Methods section.

n/a	Confirmed	
☐	☒	The exact sample size (*n*) for each experimental group/condition, given as a discrete number and unit of measurement
☐	☒	A statement on whether measurements were taken from distinct samples or whether the same sample was measured repeatedly
☐	☒	The statistical test(s) used AND whether they are one- or two-sided<br>*Only common tests should be described solely by name; describe more complex techniques in the Methods section.*
☒	☐	A description of all covariates tested
☐	☒	A description of any assumptions or corrections, such as tests of normality and adjustment for multiple comparisons
☐	☒	A full description of the statistical parameters including central tendency (e.g. means) or other basic estimates (e.g. regression coefficient) AND variation (e.g. standard deviation) or associated estimates of uncertainty (e.g. confidence intervals)
☐	☒	For null hypothesis testing, the test statistic (e.g. *F*, *t*, *r*) with confidence intervals, effect sizes, degrees of freedom and *P* value noted<br>*Give P values as exact values whenever suitable.*
☒	☐	For Bayesian analysis, information on the choice of priors and Markov chain Monte Carlo settings
☒	☐	For hierarchical and complex designs, identification of the appropriate level for tests and full reporting of outcomes
☒	☐	Estimates of effect sizes (e.g. Cohen's *d*, Pearson's *r*), indicating how they were calculated

Our web collection on statistics for biologists contains articles on many of the points above.

Software and code

Policy information about availability of computer code

Data collection	Code for this study is publicly available at https://zenodo.org/record/6122533#.YhTlw4qZOUk
Data analysis	A description of all software and settings used in this manuscript is detailed within the methods section. In brief, the main softwares used in the analysis are: CRISPRCasFinder (v4.2.19; CRISPRCasFinder v2.0.2); CRISPRDetect; CRISPRCasTyper; CRISPRMap; CRISPRloci rtracklayer package (v1.46.0); POLYAR (http://cub.comsats.edu.pk/polyapredict.htm); Cutadapt (v2.8); SAMtools (http://samtools.sourceforge.net/); bowtie; Rsamtools package (v 1.34.1); Trimmomatic (v0.38), STAR (v2.7.0a), Sambamba (v0.6.7). FeatureCounts (v1.6.0), DESeq2 (v1.24.0); IHW (v1.12.0); apeglm (v1.6.0); R package (); DESeq2 (v1.22.2); MORPHEUS (https://software.broadinstitute.org/morpheus/).

For manuscripts utilizing custom algorithms or software that are central to the research but not yet described in published literature, software must be made available to editors and reviewers. We strongly encourage code deposition in a community repository (e.g. GitHub). See the Nature Portfolio guidelines for submitting code & software for further information.

Data

Policy information about availability of data

All manuscripts must include a data availability statement. This statement should provide the following information, where applicable:

- Accession codes, unique identifiers, or web links for publicly available datasets
- A description of any restrictions on data availability
- For clinical datasets or third party data, please ensure that the statement adheres to our policy

Sequencing data that used for this analysis were published previously (see methods section). All data needed to evaluate the conclusions in the analyses are present in the text, data availability statement and/or the Supplementary Materials.

Field-specific reporting

Please select the one below that is the best fit for your research. If you are not sure, read the appropriate sections before making your selection.

☒ Life sciences ☐ Behavioural & social sciences ☐ Ecological, evolutionary & environmental sciences

For a reference copy of the document with all sections, see nature.com/documents/nr-reporting-summary-flat.pdf

Life sciences study design

All studies must disclose on these points even when the disclosure is negative.

Sample size	N/A
Data exclusions	N/A
Replication	N/A
Randomization	N/A
Blinding	N/A

Reporting for specific materials, systems and methods

We require information from authors about some types of materials, experimental systems and methods used in many studies. Here, indicate whether each material, system or method listed is relevant to your study. If you are not sure if a list item applies to your research, read the appropriate section before selecting a response.

Materials & experimental systems

n/a	Involved in the study	
☒	☐	Antibodies
☒	☐	Eukaryotic cell lines
☒	☐	Palaeontology and archaeology
☒	☐	Animals and other organisms
☐	☒	Human research participants
☒	☐	Clinical data
☒	☐	Dual use research of concern

Methods

n/a	Involved in the study	
☒	☐	ChIP-seq
☒	☐	Flow cytometry
☒	☐	MRI-based neuroimaging

Human research participants

Policy information about studies involving human research participants

Population characteristics	Eight samples of matched malignant and normal-adjacent to tumor prostate tissue from four patients with prostate cancer were obtained from the Erasmus MC tissue bank.
Recruitment	N/A
Ethics oversight	Use of the samples for research purposes was approved by the Erasmus MC Medical Ethics Committee according to the Medical Research Involving Human Subject Act (MEC-2004-261; MEC-2010-176) and patients were notified via an informed consent and asked for permission.

Note that full information on the approval of the study protocol must also be provided in the manuscript.
